# From resource to female defence: the impact of roosting ecology on a bat's mating strategy

**DOI:** 10.1098/rsos.160503

**Published:** 2016-11-02

**Authors:** Linus Günther, Marlena D. Lopez, Mirjam Knörnschild, Kyle Reid, Martina Nagy, Frieder Mayer

**Affiliations:** 1 Museum für Naturkunde, Leibniz Institute for Research on Evolution and Biodiversity, Invalidenstrasse 43, 10115 Berlin, Germany; 2 Faculty of Veterinary and Agricultural Sciences, The University of Melbourne, Werribee, Victoria, Australia; 3 Free University Berlin, Institute of Biology, Animal Behavior Lab, Takustrasse 6, 14195 Berlin, Germany; 4 Smithsonian Tropical Research Institute, Roosevelt Avenida, Tupper Building 401, Balboa, Panama; 5 University of Illinois, Department of Biological Science, Chicago, IL, USA; 6 Department of Sensor Technology, Friedrich-Alexander University Erlangen-Nuremberg, Paul-Gordan-Strasse 3/5, 91052 Erlangen, Germany

**Keywords:** male territoriality, site-dependent dominance, social dispersion, resource-defence polygyny, female-defence polygyny, *Rhynchonycteris naso*

## Abstract

With their extraordinary species richness and diversity in ecological traits and social systems, bats are a promising taxon for testing socio-ecological hypotheses in order to get new insights into the evolution of animal social systems. Regarding its roosting habits, proboscis bats form an extreme by occupying sites which are usually completely exposed to daylight (e.g. tree trunks, vines or rocks). This is accompanied by morphological and behavioural adaptations to remain cryptic in exposed day roosts. With long-term behavioural observations and genetic parentage analyses of individually marked proboscis bats, we assessed its social dispersion and male mating strategy during day and night. Our results reveal nocturnal male territoriality—a strategy which most closely resembles a resource-defence polygyny that is frequent also in other tropical bats. Its contrasting clumped social dispersion during the day is likely to be the result of strong selection for crypsis in exposed roosts and is accompanied by direct female defence in addition to male territoriality. To the best of our knowledge, such contrasting male mating strategies within a single day–night cycle have not been described in a vertebrate species so far and illustrate a possible evolutionary trajectory from resource-defence to female-defence strategy by small ecologically driven evolutionary steps.

## 1. Introduction

Animal social systems can be characterized by social dispersion (group size and spacing), mating system and natal dispersal patterns (e.g. [1–3]). As in vertebrates the investment in progeny is usually much higher in females than in males, dispersion of females is influenced by the distribution of resources (e.g. food and shelter) and risks (e.g. predation and diseases). Female dispersion in turn determines male distribution and male mating strategies [1,2,4–6]. Aggregated females open up the possibility for polygyny—the prevailing mating system among mammalian species [2,7,8]. The majority of different types of polygyny in mammals is defined by the means males use to monopolize access to females—either classical, by direct female defence (female-defence polygyny) or, uncommon in mammals, by defending a resource critical for females (resource-defence polygyny). Which strategy males use is assumed to depend on the economic defensibility of females or resources and the male's role in parental care [2,5]. Although the potential for intraspecific transitions between all four main classes of mating systems (monogamy, polygyny, polyandry and promiscuity) has been shown in several species (reviewed in [9,10]), studies on intraspecific transitions between male mating strategies (i.e. from resource defence to female defence or *vice versa*) are rare (e.g. guinea pig, *Cavia porcellus* [11]; golden-winged sunbird, *Drepanorhynchus reichenowi* [12]). They offer a valuable opportunity to gain new insights into ecological causes of male mating strategy evolution.

Bats—the second largest mammalian order—are rarely included in the debate (e.g. [1]) although their extraordinary diversity in ecological traits and social systems is promising for testing socio-ecological hypotheses and getting new insights into the evolution of animal social systems, a topic which is hitherto biased towards fish (e.g. [13,14]), birds (e.g. [12,15]), rodents (e.g. [16,17]), ungulates (e.g. [6,18]) and primates (e.g. [19,20]). Like most vertebrates, bats rely on a place to rest (i.e. roost sites), where they socially interact, mate, rear their young or hibernate [21]. Roost sites facilitate complex social interactions, give shelter from inclement weather and predation and support energy conservation [22]. Many morphological (e.g. flattened skulls, pads and discs on feet and wrists, pelage markings), physiological (e.g. torpor) and behavioural characteristics (e.g. clustering, synchronous nightly departures) are seen as adaptations for roosting and reflect compromises between body size, manner of flight, energy economy, variation in the physical environment and predation pressure [21,23]. Thus, roosting habits play an important role in the ecology and evolution of bats [21] and are discussed to also influence social organization and mating systems of bats (e.g. [22,24–26]).

While limited roost availability can act as promoter for aggregation and group living in bats (e.g. [21,22,26,27]), it is also discussed to promote a male resource-defence strategy as limited roosts form a defensible resource crucial for females (e.g. *Carollia perspicillata* [28], *Artibeus jamaicensis* [29,30], *Pipistrellus pipistrellus* [31]; reviewed for tent-roosting bats in [32]). In some tent building bats, males were found to build leaf tents, which are used by females (*Cynopterus sphinx* [33–35] and *Cynopterus brachyotis* [36]). The impact of roost types on social organization and mating strategy is indicated by bat species with intraspecific variation concerning social systems, which correlates with the use of different roost types with varying persistence. For instance, banana pipistrelles (*Neoromicia nana*) form year-round single-male and multi-female groups (harems) with male territorial defence when roosting in persistent thatches [37]. Though, if inhabiting ephemeral furled leafs males rarely roost together with females during parturition and lactation, but either solitary or with up to 11 females during other times of the year [38].

Despite the growing evidence of roost types as an important factor in the evolution of social systems of bats, our understanding of the way and degree of the influence is still scarce [22]. Derived roosting habits like perching on fully exposed structures in contrast to hiding inside concealed structures are found in a few members of emballonurid bats. This offers a unique opportunity to study the impact of an exceptional roosting ecology on the social system. Examples of roost choice in neotropical emballonurid bats include well-covered sites under fallen trees (*Cormura brevirostris* and *Peropteryx kappleri* [39–41]), constantly dry and usually shadier sites higher up on tree trunks beneath branch forks or protected by minor concavities in the bole (*Saccopteryx leptura* [42]), or sites in semi-darkened areas of huge tree buttress cavities, cave entrances, the inside of abandoned houses or shadier corners on man-made structures (*Saccopteryx bilineata* [42,43]). Proboscis bats (*Rhynchonycteris naso*) form an extreme by occupying areas which are usually completely exposed to daylight and temporarily even direct sunlight ([42]; L.G. and M.N. 2013, 2014, personal observations). Their camouflage coloration and cryptic behaviour (i.e. synchronized grooming and urinating among group members and rocking behaviour during gusts of wind to remain cryptic during motion) are interpreted as adaptation to these very exposed day roosts [40,42,44–46]. Considering these strong morphological and behavioural adaptations to remain cryptic in exceptionally exposed roosts during the day, we also expect an impact on the social system of proboscis bats.

The very small insectivorous proboscis bat (3–4 g) is distributed in lowland rainforests from southern Mexico to southern Brazil [47]. It roosts on exposed parts of tree trunks, branches, vines or man-made structures in year-round stable social groups of up to 50 individuals with males and females at about equal numbers, which space themselves at 5–10 cm from each other in day roosts. Roost sites are situated in the immediate vicinity of rivers [42], upon which *R. naso* exclusively forages. Recently, it was shown that female proboscis bats habitually disperse from their natal colony at an early age of approximately two to four months, while at least half of the male colony offspring settles in the natal colony, where some of them reproduce [48]. Reproduction usually takes place within two distinct mating periods: one seasonal mating period (SMP) at the end of the rainy season (October/November) and one postpartum oestrus mating period (PEMP) during the parturition period (April/May) approximately five months after conception ([48,49]; L.G. and M.N. 2013, 2014, personal observations). Based on behavioural observations in the day roost, the mating strategy of males has been reported to be one of direct female defence, probably with a dominance hierarchy among male group members [1,40,42,48]. With observations in the day roost, Nagy *et al*. [48] confirmed the dominance of one male in the group, but paternity analysis showed that six males successfully reproduced. Thus, previous day observations cannot fully explain the social structure and male mating success in proboscis bats.

In this study, we use long-term behavioural observations of individually marked proboscis bats in combination with genetic parentage analyses to assess social dispersion and male mating strategy during day and night. In contrast to the vast majority of other bat species, proboscis bats usually inhabit very exposed and well-lit structures [42]. Thus, we assume that the clumped roosting of mixed sex groups during the day is a derived trait and the result of selection for cryptic behaviour on exposed roost structures. At night, we hypothesize to still observe an ancestral strategy, namely that male proboscis bats establish themselves at preferred sites in their roost where they are territorial or dominant (see the electronic supplementary material, figure S2 for a sketch of this main hypothesis).

## 2. Material and methods

### 2.1. Field methods

The study was conducted between 2010 and 2014 in one colony (Cabina 5) at the La Selva field station of the Organization for Tropical Studies (Costa Rica, Province Heredia, 10°25′ N/84°00′ W). The study colony is located on the outside, under the extending roof (electronic supplementary material, figure S3) of an inhabited wooden station cabin, and thus the bats were well habituated to human presence. This roost site is known to have been inhabited by proboscis bats for at least 15 years. Mist nets (Ecotone® monofilament, Gdynia, Poland) were used to capture the bats when emerging from their roost at dusk. To prevent bats from connecting the capturing event with a potential threat to their roost, mist nets were set several metres away from the roost. Bats were marked individually with coloured plastic bands on their forearms (AC Hughes® Ltd., UK, size XCS). A small cut (around 3–4 mm) in the plagiopatagium ensured the correct fit of the bands to both forearms of the bat without moving and potentially hurting the bat's plagiopatagium. Females were banded with a unicoloured and numbered ring on the left forearm and with a bicoloured ring on the right forearm, whereas males were banded vice versa. A small tissue sample from the plagiopatagium or chiropatagium was taken (Stiefel® biopsy punch, 4 mm Ø) of each bat for genetic analysis (the resulting hole healed completely within two to four weeks). Captured bats were sexed, weighed (16 g Pesola® spring scale), and their age was determined (juvenile, subadult or adult; see [48] for details on age determination). The classes correspond to an approximate age of zero to four months for juveniles, 5–10 months for subadults and older than 10 months for adults. Details on numbers of banded and genetically sampled bats between 2006 and 2014 during this and a prior study by Nagy *et al*. [48] are provided in table 1.

**Table 1.** Number of banded and genetically sampled bats between 2005 and 2014, during the present study and a prior study by Nagy *et al*. [48].

		cabina 5		other colonies	
age	sex	banded	sampled	banded	sampled
adult	female	26	26	109	119
	male	30	29	95	101
subadult	female	32	32	38	38
	male	15	14	24	24
juvenile	female	30	33	25	32
	male	38	40	19	31
total		171	174	310	345

#### 2.1.1. Census observation

Exposed roosting habits and the possibility to approach the bats up to 5 m permitted us to detect and, if banded, identify all present bats in the roost during day and night observations. We determined number and identity of banded bats, number of unbanded bats and motherhood of pups by nursing observations of banded mothers and pups with binoculars and digital pictures. In addition, we determined the exact location of the bats based on a grid with approximately 1 × 1.4 m^2^ (see crossbeams, electronic supplementary material, figure S3). Night census observations were carried out after the first foraging period when the bats had returned to the roost to rest (between 1 and 8 h after they had left the roost at dusk). To locate the bats in the dark, dimmed light was used and as few photos with flash as possible were taken to determine the individual colour combination of the banded bats. Census observations were conducted on a daily to at least weekly basis during mornings (between dawn and noon) and afternoons (between noon and dusk) throughout the following periods: March–December 2013 (137 mornings, 88 afternoons), April–November 2014 (121 mornings, 87 afternoons) and at random day times during the following periods: July–August 2010 (14 days), April–May 2011 (32 days), July 2011 (14 days) and in July 2012 (21 days). In total, 152 night census observations were conducted during the following periods: September 2010 (7 nights), April–May 2011 (19 nights), May 2013 (2 nights), October–December 2013 (13 nights), April–November 2014 (118 nights).

#### 2.1.2. Behavioural observations during day

We recorded all behavioural interactions among bats of the two social groups in the study colony (see results for a definition of groups) during 592:36 h (focal group one) and 532:46 h (focal group two; ad libitum sampling *sensu* Altmann [50]). Behavioural interactions were monitored during the following periods: April–December 2013 (378:03 h during 162 days in group 1; 346:28 h during 163 days in group 2), April–May 2014 (108:43 h during 42 days in group 1; 90:02 h during 42 days in group 2), September–November 2014 (103:58 h during 42 day in group 1; 95:31 h during 42 days in group 2). On average, we observed each social group for 2–3 h d^–1^ and observation sessions were evenly distributed across daytime. During focal group observations, it was possible to observe all group members at the same time because all group members clustered within small assessable areas (approx. 1–3 m^2^). Owing to *R. naso's* spacing behaviour (5–10 cm individual distance to each other), all physical interactions or individual approaches between group members were fairly easy to detect and observe.

#### 2.1.3. Behavioural observations during night

At night, a constant observation of the whole social groups was not possible, because the groups split up between different sites. Thus, night observations were focused on specific sites within the roost, where only a fraction of the whole group was roosting. These focal site observations were conducted during an overall period of 23:02 h in 20 individual nights between September and November 2014. The focal sites were illuminated with two LED-infrared spotlights, recorded with a night vision camera (Bell & Howell DNV16HDZ Full HD Rouge), and bats were simultaneously monitored on the video camera screen. Parallel to this setting a DSLR camera was used to capture the coloured bands and allow individual identification of bats at the beginning of each recording session and each time a new bat arrived. In addition to focal site observations, the whole roost was constantly checked for behavioural interactions during an overall period of 24:32 h throughout 12 nights in October and November 2014. During that time, behavioural observations were carried out by constantly scanning the extending roof back and forth with an analogue night scope (Yukon 3 × 50 Exelon). If an interaction was observed, a photo was taken to determine the involved individuals.

### 2.2. Census analyses

Based on census observations, we calculated fidelity indices *F sensu* Heckel *et al*. [51]. First, based on the constant usage of the same areas of the roost by the same groups of bats, we calculated individual fidelity to a social group (*F*_group_). *F*_group_ was calculated during day and night and corresponds to the proportion a bat was observed in the area of a group in relation to its absolute presence in the roost. Second, individual fidelity of adult bats to a certain site in the roost (*F*_site_) was calculated based on the number of observation events and corresponds to the proportion a bat was present at a certain site in the roost between its first and last day of observation. *F*_site_ was calculated for morning, afternoon and night separately.

Fidelity indices were calculated for the SMP, the PEMP and the non-mating period (NMP) separately. Start and end of the two annual mating periods were determined based on the first and last observed copulation attempt. This resulted in the following distribution of census events during the different periods: NMP 2010 (July–September): 14 days, 7 nights; PEMP 2011 (April–May): 32 days, 19 nights; NMP 2011 (July): 14 days; NMP 2012 (July): 21 days; PEMP 2013 (March–May): 33 mornings, 23 afternoons, 2 nights; NMP 2013 (May–October): 53 mornings, 41 afternoons; SMP 2013 (October–November): 40 mornings, 21 afternoons, 13 nights; PEMP 2014 (April–May): 41 mornings, 22 afternoons, 44 nights; NMP 2014 (May–September): 41 mornings, 34 afternoons, 30 nights; SMP 2014 (October–November): 32 mornings, 26 afternoons, 44 nights. As the timing of day census events between 2010 and 2012 was not registered, statistics on site fidelity during morning and afternoon is based on data only from 2013 and 2014.

Only individuals that were adult during the respective observation period and present at least until the end of the period in which they had been banded were included in fidelity calculations (*n* = 22 females; *n* = 22 males). Bats were not included if they disappeared before the end of the period of their banding (*n* = 6 females and *n* = 4 males) because their disappearance was probably caused by disturbance. Individuals with a day roost fidelity below 0.5 during the respective period (*n* = 1 females and *n* = 6 males) were not considered as members of the social groups and were thus not included in the calculations. We also excluded individuals from calculations that were juvenile (*n* = 27 females and *n* = 31 males) or subadult (*n* = 20 females and *n* = 20 males) during the respective period.

### 2.3. Behavioural analyses

For this study, we defined three different behavioural interactions in the context of mating: copulations, copulation attempts rejected by females and female-defence actions performed by males. Copulations started with a male approaching a female from behind, subsequently mounting the female's back until their heads were almost at the same level. A copulation was considered to be successful if we observed the male flattening its interfemoral membrane, giving several short thrusts and finally tapping the female with his chin on her back and retracting from the female voluntarily. Copulation attempts also started with a male approaching a female from behind and mounting the female's back but were rejected by the female hitting the male with a wing and/or by flying/crawling away. The third category ‘female-defence action’ comprises different scenarios. A male was regarded to have performed a female-defence action, if he successfully chased away another male that was closely perching behind a female, approaching a female or attempting to mount a female. This defence was either achieved by quickly approaching the couple and/or hitting the male with its wing, prompting the male to crawl or fly away. In addition, a male that was perching closely behind a female or trying to mount a female and successfully defended his position against another male that attempted to take his position was also regarded to have performed a female-defence action.

### 2.4. Genetic analyses

Ethanol (80%) was used to preserve tissue samples, and the salt–chloroform procedure of Miller *et al*. [52] modified by Heckel *et al*. [51] was used for DNA isolation (for details on PCR conditions, see [53,54]). We used the DNA Analyser 4300 (LI–COR®; Biosciences) and the SAGA^GT^ (LI–COR®; Biosciences) allele scoring software to genotype a total of 156 individuals (*n* = 40 adults, *n* = 46 subadults, *n* = 70 juveniles) caught between 2012 and 2014 at the colony ‘Cabina 5’ and two other study colonies nearby at 10 highly polymorphic microsatellite loci [53,54]. All individuals were genotyped at least at eight loci, and genotypes were 99.7% complete. See electronic supplementary material, table S1 for allele numbers per locus, results of Hardy–Weinberg tests, null allele frequencies, and non-exclusion probabilities for the 10 microsatellite markers. A total of 354 individuals, which were caught in the study colony and 14 additional colonies in that area between 2005 and 2011 and genotyped by the authors in a prior study with same methods and protocols were included in the following parentage analyses [48].

Parentage analyses was performed with CERVUS v. 3.0 [55] for 87 potential colony offspring caught between May 2011 and November 2014, consisting of 58 bats caught as juveniles and 29 bats caught as subadults in the study colony Cabina 5. Maternity analyses were carried out with all 10 loci, while one *x*-linked gonosomal locus (Rn16) was left out for male–male paternity assignments [54]. Maternity of 49 *R. naso* caught as juveniles was determined by nursing observations and confirmed with genetic analysis. For six juvenile and 22 subadult individuals maternity was analysed entirely with CERVUS v. 3.0 [55]. All males caught as adults between 2006 and 2014 (*n* = 126) and resident males that matured prior to the respective mating period when potential colony offspring were conceived (*n* = 36) were treated as putative fathers for paternity assignment of pups with known (*n* = 59) and with unknown mothers (*n* = 28). Simulations were run with 100 000 cycles, a proportion of 80% sampled candidate fathers, an estimated genotyping error of 2.2% (estimated with CERVUS v. 3.0 and based on 22 mismatches between 80 known mother offspring pairs from the prior study by Nagy *et al*. [48] and this study), and a proportion of 14.5% candidate fathers that were relatives, related to the true father by *r* = 0.5. Although on average we had sampled 98% of the individuals in the study colony, we attempted to account for possible extra-colony paternities by choosing a lower sampling rate of 80% candidate fathers. The percentage of relatives among candidate fathers was estimated by Nagy *et al*. [48] based on the results of the kinship analysis between adult males. Simulations were performed for two confidence levels (80% and 95%). One mismatch per parent–offspring dyad was accepted, thus two independent mismatches between an offspring and each of its parents to account for genotyping errors. Fifty-six parent pairs were assigned at 95% confidence, and one parent pair was assigned at 80% confidence. For two female subadult immigrants, only the mother was assigned at 95% confidence, and in one case where the mother was not genetically sampled only the father was assigned at 95% confidence.

In contrast to all other loci, the locus Sb85 showed significant evidence for null alleles (electronic supplementary material, table S1). Therefore, we analysed maternity with and without the locus Sb85 for the whole dataset from 2006 to 2014. With the locus Sb85, 145 mothers–offspring pairs were assigned at 95% confidence. The analysis without Sb85 resulted in 10 additionally assigned mother–offspring pairs and four differently assigned mother–offspring pairs. In 12 of these 14 differences, the locus Sb85 showed no evidence for null alleles since the candidate offspring was heterozygous; therefore, we used results from parentage analysis with all loci (see also [48]).

### 2.5. Other statistical analyses

Other statistical analyses besides parentage analyses were performed with R v. 3.2.1 [56]. The Shapiro–Wilk normality test was used to test for normal distribution. For examining the median difference of two non-normally distributed datasets, the two-tailed Mann–Whitney *U*-test with continuity correction was used. Medians are presented with interquartile range (IQR) in the form of first quartile to third quartile (i.e. IQR = Q_1_–Q_3_). Whiskers in boxplots show Q_1_ minus 1.5 times IQR or Q_3_ plus 1.5 times IQR, respectively. Outliers are defined as values beneath or above the ‘1.5 cut-off’.

## 3. Results

Up to 31 male (range = 20–31) and 26 female (range = 23–26) proboscis bats roosted under the eaves of a large house at La Selva Biological Station, Costa Rica. For details on number and distribution of age classes during the different periods, see table 2. Bats formed two to five clusters that used five spatially separated sites on three sides of the house (electronic supplementary material, figure S3). Bats belonging to one cluster perched with approximately 5–10 cm average individual distance to each other during the day and slightly higher individual distance of approximately 5–100 cm at night. Depending on the choice of sites in the roost, distances between the clusters were approximately between 6 and 20 m during the day and approximately between 2 and 6 m at night. Hence, the distance between two adjacent clusters was always at least two orders of magnitude larger (day time) or double (night time) than the distance between the members within each cluster. In the five-year study period (2010–2014), we individually marked a total of 59 males (18 adult, 7 subadult and 34 juvenile) and 66 females (14 adult, 23 subadult and 29 juvenile) of the study colony. Additionally, five adult females and seven adult males banded in a prior study by the authors were still present in the roost during the study. Throughout the main observation period in 2013 and 2014, all colony members were individually marked and genetically sampled.

**Table 2.** Details on age classes and group composition of the two social groups (Gr.1 and Gr.2) in the roost ‘Cabina 5’ in 2013 and 2014 during PEMP, NMP and SMP. The category ‘Presence < 0.5’ comprises short-term visitors and individuals that were present less than half of the census events of the regarding period. At the end of each period, all individuals were banded.

		2013						2014					
		PEMP		NMP		SMP		PEMP		NMP		SMP	
age	sex	Gr.1	Gr.2	Gr.1	Gr.2	Gr.1	Gr.2	Gr.1	Gr.2	Gr.1	Gr.2	Gr.1	Gr.2
adult	**♀**	9	7	9	7	8	7	6	7	10	7	11	6
	**♂**	7	6	9	7	9	7	10	5	11	6	6	5
subadult	**♀**	0	0	0	0	3	2	6	1	2	2	0	1
	**♂**	0	0	3	1	6	4	4	2	1	2	1	3
juvenile	**♀**	4	3	3	1	2	1	2	2	1	1	1	1
	**♂**	3	4	6	5	3	3	3	4	1	1	1	1
presence < 0.5	**♀**	0	0	1	2	2	0	0	2	0	0	0	0
	**♂**	0	0	0	0	0	0	0	0	4	1	3	0
unbanded group members		0	0	0	0	0	0	0	0	0	0	0	0
total		23	20	31	23	33	24	31	23	30	20	23	17

### 3.1. Group fidelity (*F*_group_)

**Figure 1.**
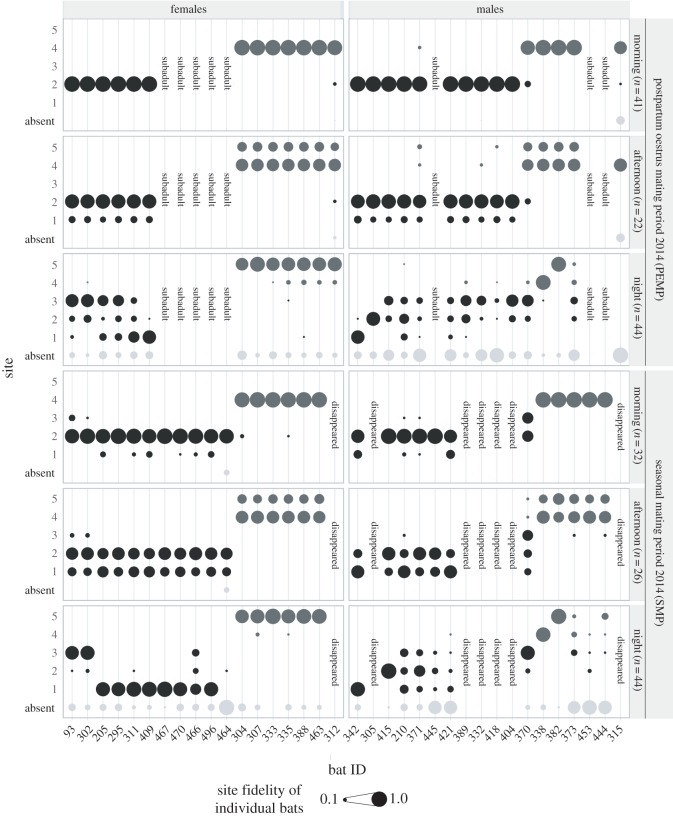
Individual fidelity to the five different sites in the study roost in PEMP and SMP 2014 during morning, afternoon and night. Light grey dots indicate the proportion of absence from the roost. The size of each dot reflects the exact site fidelity or proportion of absence and adds up to one for each individual in each box. Black dots indicate the individual presence in group 1, dark grey dots the individual presence in group 2. The number of census events during morning, afternoon and night is given in brackets. Subadult bats that became adult during 2014 are labelled as such. Individuals with the status ‘disappeared’ died or left the colony without returning until the end of the study.

We defined two social groups within the colony based on the observation of constant usage of the same sites within the roost by the same set of bats over each observation period (figure 1; group 1 in black, group 2 in dark grey). Members of group 1 usually roosted in sites 1–3 and members of group 2 usually occupied sites 4 and 5 during day and night (figure 1). See table 2 for details on group size, sex and age distribution. Our distinction of two social groups was supported by high fidelity indices for both female and male adults towards their groups during day (median *F*_group_ females = 1.00, IQR: 1.00–1.00; median *F*_group_ males = 1.00, IQR: 1.00–1.00) and night (median *F*_group_ females = 1.00, IQR: 1.00–1.00; median *F*_group_ males = 0.97, IQR: 0.85–0.99); though, at night individual group fidelity showed higher interindividual variation and lower values among males compared with females. This was consistent throughout all observation periods.

### 3.2. Site fidelity (*F*_site_)

In the following three paragraphs, the pattern of individual site fidelity in the study colony is described. See figure 1 for an exemplary visualization of the pattern during PEMP and SMP 2014. Detailed individual values of all periods (2010–2014) are provided in the electronic supplementary material, tables S4–S6. Each group mainly used two sites within the roost. Group 1 (figure 1; black dots) used the site on the left front of the house (site 2) and a shadier site around the corner on the left site of the house (site 1). Group 2 (figure 1; dark grey dots) used the site on the right front of the house (site 4) and a shadier site around the corner on the right site of the house (site 5). A fifth site, which lay in the front centre of the roof (site 3) was occasionally used by members of group 1 during days of the SMP and frequently at night by females of group 1 and males of both groups.

#### 3.2.1. Site fidelity during morning (figure 1, rows 1 and 4)

Females and males of both groups showed high fidelity towards a single roosting site throughout the morning (fidelity to the preferred site = max *F*_site_). Group 1 roosted at ‘site 2’ and group 2 at ‘site 4’. This was consistent throughout all observation periods (median max *F*_site_ females = 0.97, IQR = 0.93–0.99; median max *F*_site_ males = 0.94, IQR = 0.90–1.00). However, during the SMP group 1 occasionally split up between ‘site 1’, ‘site 2’ and ‘site 3’, resulting in a lower site fidelity of the group members to the main site ‘2’ and higher interindividual variation among males and females (SMP: median max *F*_site_ group 1: females = 0.88, IQR = 0.81–0.94; males = 0.87, IQR = 0.78–0.95; *n*_females_ = 13, *n*_males_ = 13).

#### 3.2.2. Site fidelity during afternoon (figure 1, rows 2 and 5)

Females and males of both groups still predominantly used the same sites as during the morning. However, the entire groups relocated to a second site (group 1 to site 1 and group 2 to site 5) around the corners of the house more often than in the morning, resulting in a lower fidelity to the preferred site in both groups (median max *F*_site_ throughout all periods: females = 0.66, IQR = 0.64–0.70; males = 0.69, IQR = 0.64–0.74; *n*_females_ = 22, *n*_males_ = 23). In these cases group 1 changed from ‘site 2’ to ‘site 1’ and group 2 from ‘site 4’ to ‘site 5’. However, during NMP 2014 group 1 still showed high site fidelity towards ‘site 2’ even during the afternoon.

#### 3.2.3. Site fidelity during night (figure 1, rows 3 and 6)

Both groups were found to split up between several sites during the night. Obvious differences were found between males and females. Throughout all periods, females less frequently used the main day site of their group during night (‘site 2’ or ‘site 4’, respectively) and showed high individual site fidelities towards ‘site 1’, ‘site 5’ or ‘site 3’ respectively. Males showed larger interindividual differences, especially during the two mating periods (PEMP: median max *F*_site_ females = 0.70, IQR: 0.59–0.77; median max *F*_site_ males = 0.43, IQR: 0.18–0.67; *n*_females_ = 13, *n*_males_ = 15; SMP: median max *F*_site_ females = 0.82, IQR: 0.77–0.92; median max *F*_site_ males = 0.46, IQR: 0.17–0.77; *n*_females_ = 21, *n*_males_ = 21; NMP: median max *F*_site_ females = 0.60, IQR: 0.47–0.77; median max *F*_site_ males = 0.30, IQR: 0.20–0.60; *n*_females_ = 17, *n*_males_ = 17).

Furthermore, for each of the four main sites we found only one male with very high site fidelity (figure 1, rows 3 and 6; max *F*_site_ males during mating periods = 0.69–1.00; *n* = 13 territorial males). These males were rarely or never observed at multiple sites in the roost at night. This pattern was less distinct during NMP (max *F*_site_ males during NMPs = 0.63–0.90; *n* = 4 territorial males). Subsequently, we refer to these males as territorial males. The other males—referred to as non-territorial males—switched among sites and used two to four different sites in the roost, therefore had lower site fidelity at each site (figure 1, rows 3 and 6; max *F*_site_ = 0–0.6; *n* = 18 non-territorial males). For ‘site 4’ in PEMP and SMP 2013, we found two territorial males (ID217 and ID338). This might reflect a period of overlapping in a transition from one territorial male to another since after SMP 2013 the older male of the two disappeared, while the younger male remained territorial at the site until the end of the study. At ‘site 3’ only in PEMP 2011 and SMP 2014, a territorial male could be identified, as during other periods several males showed high fidelities to that site.

Throughout the study, we determined 13 different territorial males. During 2013 and 2014 with census data on four continuous mating periods, mean minimum tenure as territorial male was 2.4 mating periods (*n* = 8 territorial males; range: 1–4 mating periods). However, owing to the lack of information on start (*n* = 3), the end (*n* = 3) or both (*n* = 2) of the males' territorial status, actual average male tenure as territorial surely exceeds our estimates.

### 3.3. Mating behaviour

#### 3.3.1. During day

**Figure 2.**
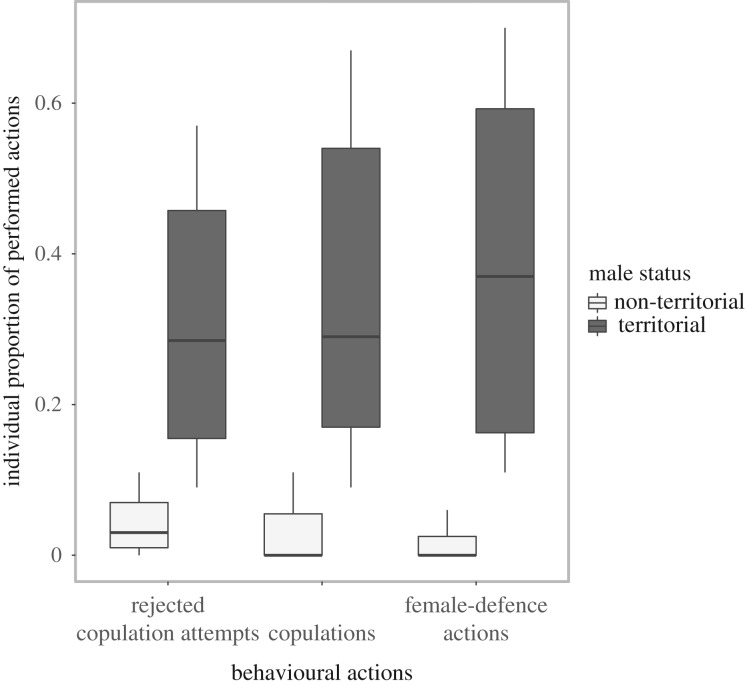
Individual proportional distribution of mating-related behavioural interactions by territorial (*n* = 8) and non-territorial (*n* = 18) males (*n* = 763 copulation attempts rejected by females, *n* = 122 copulations, *n* = 138 female-defence actions) during day. Individual proportions were calculated separately per social group and mating period. Values were averaged for males with the presence in multiple mating periods. Note that three males are included as non-territorial and territorial males in the statistics, since they became territorial between 2013 and 2014.

Observations during mating periods in 2013 and 2014 resulted in a total of 122 copulations, 763 copulation attempts rejected by females and 138 female-defence actions performed by males (see Material and methods for definition of female-defence actions). The same males which were determined as territorial at night (four to five different males per observation period and depending on the mating period) performed the majority of all rejected copulation attempts (66.7%, *n* = 513), copulations (86.8%, *n* = 105) and female-defence actions (88.4%, *n* = 122). Performances of the remaining actions were done by seven to nine of the 7–12 present non-territorial males. Individual proportion of performances in all categories per group and mating period was significantly higher for males which were determined as territorial at night than non-territorial males (figure 2; Mann–Whitney *U*-test: *n*_non-territorials_ = 18, *n*_territorials_ = 8; rejected copulation attempts: *U* = 2130.5, *p* < 0.001; copulations: *U* = 1928.5, *p* < 0.001; female-defence actions: *U* = 1870.5, *p* = 0.003). In addition, territorial males performed all daytime copulations at the site where they were also determined as being territorial at night (100%, *n* = 105) as well as the vast majority of rejected copulation attempts (91.8%, *n* = 456) and female-defence actions (82.6%, *n* = 114). In other words, if the group perched on site A, where male A was determined as territorial at night, male A was the most successful male in the group regarding the three mating-related categories during day. However, if the same group moved to site B, where male B was determined as territorial at night, male B was the most successful male in the group during day and male A stopped performing. Note that three males were included as both non-territorial and territorial males in statistics, since they became territorial between 2013 and 2014.

#### 3.3.2. At night

In SMP 2014, we conducted focal night observations of the five territories. We observed a total of 24 copulations, six rejected copulation attempts and no female-defence actions. All copulations and copulation attempts were performed by the five territorials within their own territories. Nine non-territorial males performed none of these actions. In eight cases, females left their preferred roosting site to visit another territorial male of her group in his territory for copulation. Furthermore, we have evidence of one territorial male chasing another male out of his territory without any females present (*n* = 3 observations).

### 3.4. Parentage

We were able to genetically assign a mother to 53 juveniles and six subadults of 87 analysed potential colony offspring (*n* = 58 juveniles and *n* = 29 subadults). The 28 remaining individuals without assigned mother (*n* = 5 juveniles and *n* = 23 subadults) can be regarded as immigrants as all but one of the potential colony mothers were sampled and genotyped. In addition, 24 of the 28 individuals without assigned mother were also observed to immigrate into the colony as subadults (*n* = 20), or juveniles (*n* = 4), because offspring dispersal takes place very early at an age of approximately two to four months, when some bats are still classified as juveniles.

Of 59 offspring with a genetically assigned mother, 56 can be regarded as colony offspring as they were either observed to be nursed in the colony (*n* = 49) or the mother was at least present in the social group and season the pup was sampled (*n* = 7). The mothers of the three remaining offspring (subadult females) were constant members of a different nearby study roost. Thus, these subadult females can be regarded as immigrants. In addition to the 56 genetically identified colony offspring, one pup without an assigned mother was nursed by a female which was not genetically sampled.

We determined paternity for all 57 colony offspring sired in six mating periods (*n* = 12 in SMP 2010, *n* = 5 in SMP 2011, *n* = 14 in SMP 2012, *n* = 12 in PEMP 2013, *n* = 11 in SMP 2013 and *n* = 3 in PEMP 2014). The 57 colony offspring were fathered by 17 different males. Sixteen of the 17 fathers were members of the social group their respective offspring was born in. The sole external father was banded and observed in a different nearby roost.

### 3.5. Siring success of territorial and non-territorial males

**Figure 3.**
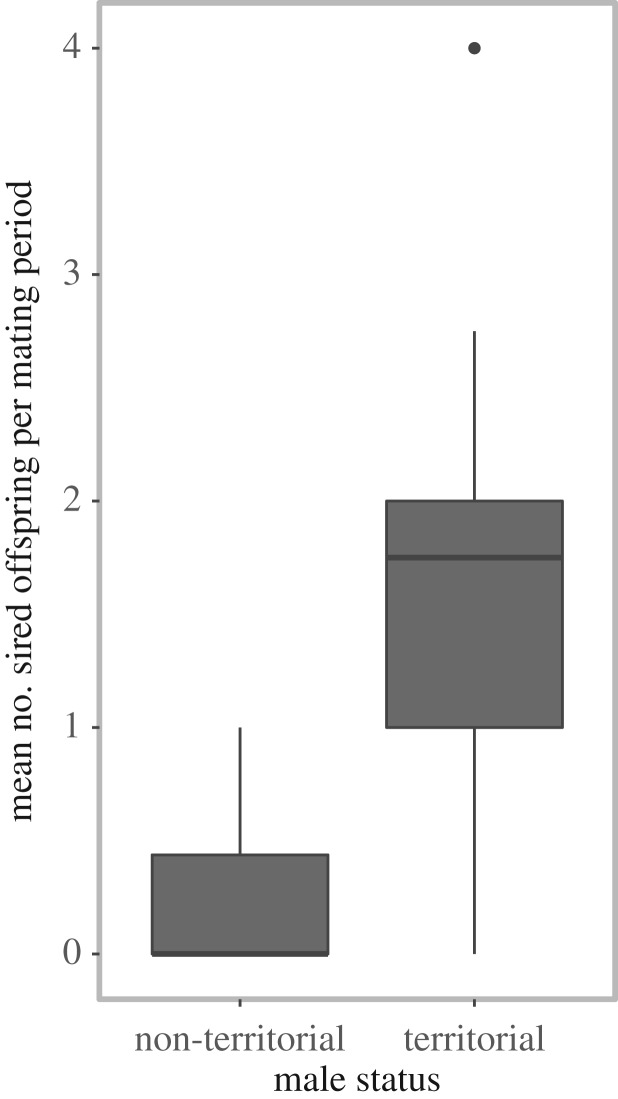
Mean number of sired colony offspring (total *n* = 52 offspring) per mating period by 32 non-territorial males (*n* = 14 offspring) and nine territorial males (*n* = 38 offspring). The number of sired offspring by each male is averaged over all mating periods the male was present.

We possess data on male territoriality for fathers of 52 colony offspring from five different mating periods either gathered during time of conception (*n* = 26 offspring) or gathered four months after conception (i.e. during the following parturition period, *n* = 26 offspring). During these five mating periods, nine of nine territorial males sired 38 of the 52 colony offspring (73.1%), while eight of 21 non-territorial males sired the remaining 14 offspring (26.9%). Median individual reproductive success per mating period was significantly higher for territorial than non-territorial males (figure 3; non-territorial males = 0.00, IQR = 0.00–0.44; territorial males = 1.75, IQR = 1–2; Mann–Whitney *U*-test: *n*_non-territorials_ = 21, *n*_territorials_ = 9, *U* = 19.5, *p* < 0.001). Two males are included as non-territorial and territorial males in the statistics, as they became territorial between the mating periods when the sampled offspring was conceived.

## 4. Discussion

Our observational and genetic results reveal striking differences between day and night regarding social dispersion in the roost and the males' main mating strategy. At night, territorial males occupy a territory. Some territories permanently include females, whereas other territories are only occasionally visited by females. At four to five sites of the study roost, we found for each site only one male that showed very high site fidelity (max *F*_site_ = 0.69–1.00). All other colony males were either absent at night or roosted at two to four different sites in the roost, resulting in low site fidelities per site (max *F*_site_ = 0.00–0.63); we refer to these males as non-territorial males. All definitions of territoriality are variants of three main themes: defended area, site-specific dominance and exclusive area (reviewed in [57]). While the first two criteria are based on behavioural interactions, the latter can be seen as the outcome of behavioural interactions [58]. In general, several operational pitfalls can occur by using behavioural interactions to define territoriality [59], but the prevailing pitfalls with most bat species and many other taxa are high mobility, nocturnal activity and complex ultrasound vocal communication that may be difficult to relate to the behavioural context. At night, territoriality in *R. naso* comes closest to an exclusive area, suggested by the exclusively high site fidelity of only one male per site. This is also supported by the fact that only males with high site fidelity, i.e. territorial males, were observed copulating at night and they exclusively copulated at their territory. Furthermore, the evidence of one territorial male chasing another male out of his territory without any females present (*n* = 3 observations) seem to represent physical interactions among males (defence actions) and might be rare escalations of the very abundant acoustical interactions. Finally, the median proportional individual reproductive success within each social group was significantly higher for territorial males compared with non-territorial males. Thus, our behavioural and genetic results provide evidence that *R. naso* males follow a territory-based mating strategy. According to Emlen & Oring [5], such a strategy can be linked to either a resource defence or a lek system. In *R. naso*, territories are sites within the roost, which are used year-round by females and thus may be considered a crucial resource. Regardless of the question whether a particular site in the roost represents an important resource for females and thus gives males the possibility to guard and defend it, the high and long-term presence of territorial males at a single site in the roost (i.e. high site fidelity) may also indicate important male characteristics to females (e.g. assertiveness and efficient foraging). The observation that females left their preferred site and former mating partner (i.e. the territorial male at that site) to visit other territorial males at other territories for copulation supports the idea that female choice occurs in *R. naso*. Based on nocturnal observations and male mating success, in *R. naso* the prevailing social dispersion and mating system at night most closely resemble mating territories in a resource-defence polygyny (*sensu* Emlen & Oring [5]). Although we have never observed any female-defence actions at night, we cannot exclude the strategy of direct female defence by *R. naso* males at night. However, our observations show that direct female defence plays only a minor role in male mating strategy at night.

By contrast, during day *R. naso* lives in cohesive groups of multiple males and females. Entire groups occasionally moved between different sites within the roost. These sites correspond to the territory sites identified at night. During day and night, territorial males were most successful regarding copulations, and territorials performed by far most copulation attempts and female-defence actions but only within their own territory. During the day, non-territorial males attempted to copulate at any site, but were almost always interrupted by the territorial male of the respective territory (i.e. direct female defence). Territorial males rarely attempted copulation outside their territory. Thus, a male can be a dominant individual at one site (in his territory), but a non-dominant individual outside of his territory within the same social group. This resembles a rare form of territoriality called site-specific dominance (*sensu* Kaufmann [60]), where a territory is defined as an area in which one individual has priority of access to a resource over other individuals who have this privilege at other areas, achieved by social interaction. Hence, based on our diurnal observations *R. naso* groups can be classified as multi-male/multi-female groups with site-specific dominance relationships among male group members and direct female defence (female-defence polygyny *sensu* Emlen & Oring [5]).

To the best of our knowledge, such contrasting classifications regarding social dispersion and male mating strategy within a single day–night cycle have not been described yet for any mammal or vertebrate species. However, intraspecific variation in mammalian and vertebrate social systems among different populations or over longer time periods (seasons) is widespread. These include variation in spatial patterns (e.g. [61]), group size (e.g. [62,63]) and mating systems [17]. Several ecological variables have been identified to correlate with variation in social systems (resource abundance, competition for food, predation pressure, population density, habitat saturation; reviewed in [9,10]). For instance, striped mice (*Rhabdomys pumilio*) in the arid succulent Karoo live in groups of multiple male and female adults in one territory, whereas male and female individuals form solitary territories in resource-rich moist grasslands [61]. Larger groups are formed if predators are present as, for example, in eastern grey kangaroos (*Macropus giganteus*) when red foxes are present [62] or in musk ox when wolf densities are increased [64]. In Alaska moose (*Alces alces gigas*), group size positively correlated with distance from cover [65].

The daily shift between a resource-defence and a female-defence polygyny offers the unique opportunity to infer the evolutionary transition between two supposedly different male mating strategies. *Rhynchonycteris naso's* nocturnal territorial structure is a trait shared among many bat species that live in multi-male/multi-female colonies (e.g. tropical bats: *Neoromicia nana* [37]; *Pteropus giganteus* [66]; *P. poliocephalus* and *P. scapulatus* [67,68]; *Miniopterus minor* [69]; e.g. temperate bats: *Tadarida brasiliensis* [70]; *Rhinolophus ferrumequinum* [71]; *Macrotus californicus* [72]; *Myotis myotis* reviewed in [73]). This is also the case for a close relative of *R. naso,* the greater sac-winged bat (*Saccopteryx bilineata*). Colonies of *S. bilineata* consist of one to several territorial (harem) males defending roosting space in the day roost. Each harem includes one harem male and on average two to three females but no other adult males [42,74,75]. The common occurrence of a resource-defence strategy among bats including *S. bilineata* suggests that this strategy represents the plesiomorphic behaviour.

The vast majority of echolocating bats occupy concealed day roosts inside of trees, leaf tents, caves, rock crevices or at least hidden areas of these structures (reviewed in [22]). By contrast, proboscis bats roost on exposed parts of tree trunks, vines, rocks or man-made structures. This roosting behaviour offers them a great variety of roost sites (e.g. also close to their obligatory foraging sites above rivers), while many other bat species are likely to compete for limited concealed or hidden roost sites (e.g. [21,22,26,27,76]). Proboscis bats inhabit roosts that are usually completely exposed ([42]; L.G. and M.N. 2010–2014, personal observations). This most probably results in a much higher predation risk in proboscis bats than in other bat species. As part of their camouflage, proboscis bats remain totally motionless during almost the whole day while rocking, grooming, stretching, urinating and behavioural interactions are almost exclusively restricted to gusts of wind [46]. This suggests that males are also restricted in their options to prevent other males from joining their group during the day. Interestingly, territorial males tolerated the presence of other males in their territory during day, although copulation attempts from other males during the day occurred. We assume that males only give up their camouflage to interact with other males if it is worth it—e.g. to prevent a copulation by another male (i.e. female-defence action). Accordingly, predation pressure probably forces territorial males to tolerate the presence of additional males in their territory in order to maintain visual crypsis during the day, but requires territorial males to also guard and defend females directly. This highlights how an ecological factor like predation pressure may drive social dispersion and male mating strategies.

At present, we do not know how territoriality in *R. naso* is achieved or maintained. However, during approximately three weeks in August and September 2013 prior to the SMP in October, we observed a remarkable number of antagonistic male–male interactions during the day—a behaviour, which is usually only observed during mating periods. These interactions could be used to achieve or maintain territorial status and should be subject to future research. In addition, vocal communication might also play an important role in the mating strategies of males, but further studies are needed to verify this.

## 5. Conclusion

Our results on *R. naso* support the theory that the outstanding diversity of social systems in chiropterans—the second largest mammalian order—is strongly influenced by its astonishing diversity in roosting strategies [21]. By making use of very exposed day roosts, *R. naso* gained access to a large variety and number of potential day roosts that are usually not occupied by other bat species. Otherwise, access to day roost is frequently assumed to be a limiting factor for bats (e.g. [22,26,27,76]). At night, male *R. naso* adopt a presumably plesiomorphic territorial strategy, a behaviour that is frequent also in other tropical bats and most closely resembles a resource-defence polygyny. Its contrasting clumped social dispersion during the day is likely to be the result of strong selection for cryptic behaviour in the exposed roosts and requires direct defence of females in addition to male territoriality. However, with site-specific dominance, territorial males adopt a strategy that allows them to maintain primary access to females despite the need of crypsis during the day. Thus, our results on the social system of *R. naso* show how roosting ecology can shape the social dispersion and mating system of a species. Finally, the night-to-day transition from a rather classical territoriality with male resource defence to clumped mixed sex groups with site-specific dominance and direct female defence illustrates a possible evolutionary trajectory from a resource-defence mating strategy to a female-defence mating strategy by small ecologically driven evolutionary steps.

## Supplementary Material

The following additional supporting material may be found in the online version of this article: Table S1 Results from allele frequency calculations with CERVUS v. 3.0 (Kalinovski et al. 2007). Figure S2 Sketch of the main hypothesis regarding the differences in social dispersion in the roost between day and night. We assume that the clumped roosting of mixed sex groups during the day is a derived trait and the result of selection for cryptic behaviour on exposed roost structures. At night, we hypothesize to still observe an ancestral strategy, namely that male proboscis bats establish themselves at preferred sites in their roost where they are territorial or dominant. The blue bats represent males. The red bats represent females. Distances between the individual bats are true to scale, while distances between the sites are not to the scale. Figure S3 Top view of the roof of ‘Cabina 5’. The extending roof is drawn in black with its grid (1-36) and the five defined sites. Table S4–S6 Detailed census data from different periods (postpartum oestrus mating period ‘PEMP’; seasonal mating period ‘SMP’; non-mating period ‘NMP’) between 2010 and 2014

## Data accessibility

Microsatellite data of typed individuals (ID, sex, sampling location and alleles of 10 loci) is deposited at Dryad Digital Repository: http://dx.doi.org/10.5061/dryad.df878 [77].
