# Supplementary material for: From resource to female defence: the impact of roosting ecology on a bat's mating strategy
Source: R Soc Open Sci. 2016 Nov 2;3(11):160503. doi: 10.1098/rsos.160503 (PMC5180135; doi:10.1098/rsos.160503)
Supplement: The following additional supporting material may be found in the online version of this article: Table S1 Results from allele frequency calculations with CERVUS v. 3.0 (Kalinovski et al. 2007). Figure S2 Sketch of the main hypothesis regarding the differences in social dispersion in the roost betwee [file rsos160503supp1.docx]

**Table S1** Results from allele frequency calculations with CERVUS v. 3.0 (Kalinovski et al. 2007).

| Locus | No. of alleles | No. of Adult individuals | H_O_ | H_E_ | PIC | NE-1P | NE-2P | NE-PP | NE-I | NE-SI | HW | F (Null) |
| --- | --- | --- | --- | --- | --- | --- | --- | --- | --- | --- | --- | --- |
| Rn 01 | 20 | 331 | 0.9 | 0.91 | 0.902 | 0.31 | 0.183 | 0.054 | 0.015 | 0.299 | NS | 0.0044 |
| Rn 04 | 29 | 328 | 0.921 | 0.918 | 0.911 | 0.285 | 0.167 | 0.045 | 0.013 | 0.295 | NS | -0.0024 |
| Rn 09 | 18 | 331 | 0.9 | 0.896 | 0.886 | 0.347 | 0.209 | 0.068 | 0.02 | 0.308 | NS | -0.003 |
| Rn 10 | 14 | 331 | 0.831 | 0.822 | 0.804 | 0.506 | 0.334 | 0.149 | 0.049 | 0.352 | NS | -0.0086 |
| Rn 11 | 18 | 331 | 0.846 | 0.868 | 0.853 | 0.421 | 0.265 | 0.104 | 0.031 | 0.325 | NS | 0.014 |
| Rn 13 | 11 | 331 | 0.867 | 0.832 | 0.811 | 0.501 | 0.33 | 0.153 | 0.049 | 0.347 | NS | -0.0213 |
| Rn 16 | 30 | 331 | 0.465 | 0.916 | 0.909 | 0.288 | 0.169 | 0.045 | 0.013 | 0.296 | *** | 0.3282* |
| Rn 18 | 64 | 329 | 0.948 | 0.96 | 0.957 | 0.151 | 0.082 | 0.012 | 0.003 | 0.271 | ND | 0.0055 |
| Sb 84 | 20 | 329 | 0.872 | 0.889 | 0.877 | 0.368 | 0.225 | 0.077 | 0.023 | 0.312 | NS | 0.0075 |
| Sb 85 | 20 | 327 | 0.706 | 0.871 | 0.858 | 0.409 | 0.256 | 0.097 | 0.029 | 0.322 | *** | 0.1026** |

For allele frequency calculations, only individuals that were observed as adults (n=331) were used. H_O_: observed heterozygosity, H_E_: expected heterozygosity, PIC: mean polymorphic information content, NE-1P: non-exclusion probability for first parent, NE-2P: non-exclusion probability for second parent, NE-PP: non-exclusion probability for parent pair, NE-I: non-exclusion probability for identity, NE-SI: non-exclusion probability for sib identity, HW: Hardy-Weinberg equilibrium test, NS: not significant; ND: not done; ***: significant and F (Null): null allele frequency. Locus Rn16 is a gonosomal locus, thus was left out for male-male paternity assignments. We tested all loci for evidence of null alleles with the software MICRO-CHECKER v. 2.2.3 (van Oosterhout et al. 2004). There was significant evidence for the presence of null alleles at Locus Rn16* (due to its gonosomal inheritance mode) and Sb85** (see last paragraph of methods section for details on handling this locus).

Mean number of alleles per locus: 24.4

Mean proportion of loci typed: 0.9967

Mean expected heterozygosity: 0.8883

Mean polymorphic information content (PIC): 0.8768

Combined non-exclusion probability (first parent): 0.00002146

Combined non-exclusion probability (second parent): 0.00000015

Combined non-exclusion probability (parent pair): 1.593E-0012

Combined non-exclusion probability (identity): 7.805E-0018

Combined non-exclusion probability (sib identity): 0.00000868

**Figure S2** Sketch of the main hypothesis regarding the differences in social dispersion in the roost between day and night. We assume that the clumped roosting of mixed sex groups during the day is a derived trait and the result of selection for cryptic behaviour on exposed roost structures. At night, we hypothesize to still observe an ancestral strategy, namely that male proboscis bats establish themselves at preferred sites in their roost where they are territorial or dominant. The blue bats represent males. The red bats represent females. Distances between the individual bats are true to scale, while distances between the sites are not to the scale.

**Figure S3** Top view of the roof of ‘Cabina 5’. The extending roof is drawn in black with its grid (1-36) and the five defined sites.

Table S4–S6 Detailed census data from different periods (postpartum oestrus mating period ‘PEMP’; seasonal mating period ‘SMP’; non-mating period ‘NMP’) between 2010 and 2014. For each period individual age class, roost fidelity and site fidelity at the preferred site (max site fidelity) are given. The number of census events during morning, afternoon and night during each period is given in brackets. Only individuals that were present at least as subadult during the study are included. Individuals born in the colony (n=25 female; n=32 males) that left the colony before subadult age (n=19 females; n=11 males) and individuals that disappeared before the end of the period of their banding (n = 6 females; n = 4 males) are not listed.

Table S5

Table S6
